# The development and validation of dried blood spots for external quality assurance of syphilis serology

**DOI:** 10.1186/1471-2334-13-102

**Published:** 2013-02-26

**Authors:** Pieter W Smit, Thomas van der Vlis, David Mabey, John Changalucha, Julius Mngara, Benjamin D Clark, Aura Andreasen, Jim Todd, Mark Urassa, Basia Zaba, Rosanna W Peeling

**Affiliations:** 1 Leiden Cytology and Pathology Laboratory, Leiden, The Netherlands; 2 London School of Hygiene & Tropical Medicine, Keppel Street, London, WC1E 7HT, UK; 3 National Institute for Medical Research, NIMR, Mwanza, Tanzania; 4 Mwanza Intervention Trials Unit, Mwanza, Tanzania

**Keywords:** Dried blood spots, Syphilis, *Treponema pallidum*, DBS, Sensitivity, Evaluation

## Abstract

**Background:**

Syphilis causes up to 1,500,000 congenital syphilis cases annually. These could be prevented if all pregnant women were screened, and those with syphilis treated with a single dose of penicillin before 28 weeks gestation. In recent years, rapid point-of-care tests have allowed greater access to syphilis screening, especially in rural or remote areas, but the lack of quality assurance of rapid testing has been a concern. We determined the feasibility of using dried blood spots (DBS) as specimens for quality assurance of syphilis serological assays.

**Methods:**

We developed DBS extraction protocols for use with *Treponema pallidum* particle agglutination assay (TPPA), *Treponema pallidum* haemagglutination assay (TPHA) and an enzyme immunoassay (EIA) and compared the results with those using matching plasma samples from the same patient.

**Results:**

Since DBS samples showed poor performance with TPHA and EIA (TPHA sensitivity was 50.5% (95% confidence interval: 39.9–61.2%) and EIA specificity was 50.4% (95% CI: 43.7–57.1%), only the DBS TPPA was used in the final evaluation. DBS TPPA showed an sensitivity of 95.5% (95% CI: 91.3–98.0%) and a specificity of 99.0% (95% CI: 98.1–99.5%) compared to TPPA using plasma samples as a reference.

**Conclusion:**

DBS samples can be recommended for use with TPPA, and may be of value for external quality assurance of point-of-care syphilis testing.

## Background

It is estimated that the burden of congenital syphilis is large, with 1.5 million cases per year worldwide [1]. These could be prevented if all pregnant women were screened and treated with a single dose of benzathine penicillin before 28 weeks gestation [2]. Syphilis testing is usually done using laboratory based assays such as *Treponema pallidum* haemagglutination assay (TPHA), *Treponema pallidum* particle agglutination assay (TPPA), rapid plasma reagin (RPR), or enzyme Immunoassay (EIA). These tests need to be used with serum or plasma samples and require a centrifuge, shaker, and refrigeration for the reagents. They are therefore less suitable than point-of-care tests (POCT) for use in rural or remote locations. POCT screening tests for syphilis that are sensitive and specific in detecting treponemal antibodies are now available [3]. The main advantages of POCTs are that they are easy to use, can be stored at room temperature, and can be used with whole blood, collected with a finger prick. The Global Report on Preterm Birth and Stillbirth and modelling studies have identified syphilis POCTs testing and treatment as an urgent priority for reducing perinatal morbidity and mortality [4-6]. Many countries have therefore started to scale up the use of POCTs in prenatal screening programmes for syphilis, but the lack of suitable methods for external quality assurance (EQA) is a serious concern.

Most QA methods have been developed to monitor the quality of tests performed in the laboratory, and are not designed for monitoring POCT usage by healthcare workers at remote locations [7]. Dried Blood Spots (DBS) have been suggested to be a suitable EQA methodology for HIV POCTs, since they are easily collected, require minimal training and can be sent at ambient temperature for retesting at a centralised laboratory [8]. DBS samples have been used in prevalence studies for syphilis serology, but without prior validation of the methodology [9-11]. DBS samples have been evaluated with TPPA [12], TPHA [13], and an in-house EIA which is not commercially available [14]. The TPHA used in the study by Backhouse *et al.* is no longer commercially available. The fourteen year old TPPA protocol used by Coates *et al.* did not include a control for biologically reactive samples, and the final testing concentration of DBS eluate was more diluted than with plasma, potentially leading to reduced sensitivity for samples with low antibody titres [12]. The objectives of this study were to develop and validate DBS protocols for use with commercially available syphilis diagnostic assays, and to determine their performance in syphilis serological assays using plasma samples as a reference.

## Methods

### Research setting

The Kisesa open cohort is a well-established on-going community-based study covering six villages in northern Tanzania. The cohort study conducts regular demographic surveillance on HIV prevalence and incidence [15]. Sexual behaviour data and HIV status are collected by surveys for which all adults aged 15 years or older are eligible to participate. Study participants that opted for voluntary counselling and testing (VCT) were offered HIV and syphilis POCT (SD Bioline, USA) performed by trained and experienced technicians. If the VCT participant was treponemal antibody positive by POCT, free medical treatment was provided according to the Tanzanian government recommendations, and all those positive for HIV were referred to Tanzanian care-and treatment centres. The study was approved by the Medical Research Coordinating Committee of the National Institute for Medical Research in Tanzania (NIMR) and the ethical committee of the London School of Hygiene and Tropical Medicine.

### Procedures

For the community based HIV study, DBS samples (Proteinsaver 903 filter paper, Whatman, GE healthcare, USA) were collected by finger prick, air dried at ambient temperature for at least 3 hours and stored with desiccants in individual ziplock bags. DBS samples were transported at ambient temperature and upon arrival at the laboratory stored at −20°C. After participants donated a DBS sample, they were invited to opt in for VCT. Whole blood samples were collected from consenting participants who opted in for VCT by trained clinicians and transported to the NIMR laboratory in Mwanza. EDTA blood samples were bar-coded in the field to ensure anonymous testing. Within 24 hours, the blood samples were centrifuged and plasma was stored in 3 aliquots (1 mL each) at −20°C. From all subjects participating in the serosurvey who opted for VCT, 1,645 samples were randomly selected for this study.

All syphilis serology assays were performed according to manufacturer’s directions. The positive and negative controls supplied with the kits were used with every run. The technicians were blinded to other test results and TPHA and TPPA results were read by two trained laboratory technicians. All tests were performed in at the NIMR laboratory, which participates in the WHO EQA programs for TPPA and Rapid plasma Reagin (RPR) tests. An active syphilis case is defined as RPR positive, confirmed by a treponemal test. The results were entered using the laboratory information management system.

**Figure 1:**
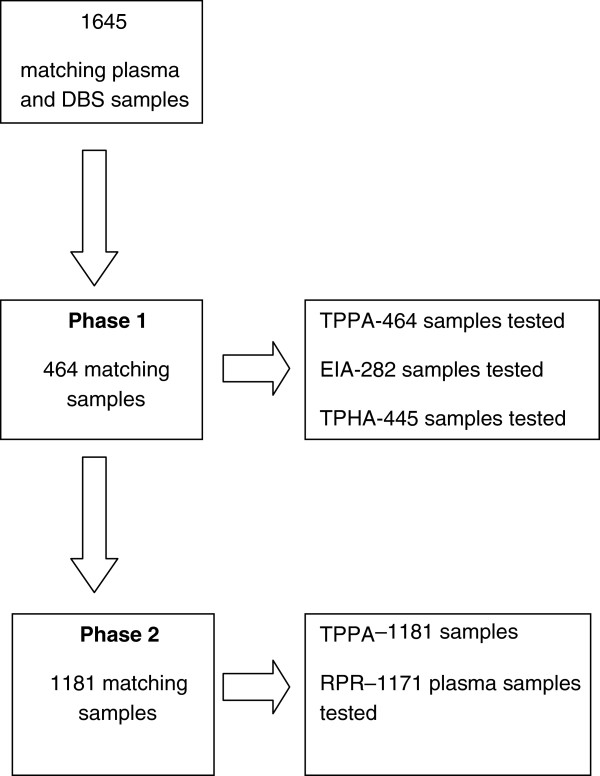
**Study design diagram.** The number of matching plasma and DBS samples used for each phase of the study are given in the boxes on the left and the number of matching DBS and plasma samples tested for each of the assays are given in the boxes on the right. RPR has been performed on plasma, not on DBS.

The protocol development and evaluation was divided into two phases. In the first phase, we determined the feasibility of using DBS with TPHA, TPPA and EIA using 464 samples. During DBS protocol development, care was taken to ensure an equivalent DBS sample input was used for each serological method, compared to plasma. In the second phase, the serological tests that work best with DBS were selected and validated with 1,181 samples (Figure 1). Plasma samples were used as the reference standard. The RPR test was performed on plasma samples that gave discrepant results between DBS and plasma samples to determine if any active syphilis cases might have been missed. Active syphilis infection was defined as a positive TPPA and RPR test. Samples were prospectively collected from July through September 2010 and were tested until March 2011. During phase 1, the preliminary evaluation of different syphilis serological methods with DBS samples, TPPA, TPHA and EIA were all performed from the same DBS eluate as only one DBS spot was available. As a reference method, TPPA, TPHA and EIA were performed on matching plasma samples in parallel. To ensure blinded reading in the laboratory, plasma samples were tested before DBS samples. Additionally, the three tests on DBS were performed separately from each other, to maintain blindness to other test results.

### TPPA

A total of 1,645 plasma aliquots were brought to room temperature and 25 μl were used to test for treponemal antibodies by TPPA (Fujirebio, Tokyo, Japan), according to manufacturer’s protocols, by laboratory technicians who routinely perform TPPA on plasma samples.

For DBS TPPA testing, the protocol was adjusted as follows. A 6 mm disk was manually punched and eluted in 100 μl Phosphate buffered Saline (PBS) with 0.05% Tween80 in a clean 96 flat wells plate, shaken for 2 minutes and eluted overnight at 4°C. Upon the next day, the plate was shaken 2 minutes and brought to room temperature. 25 μl sample dilution buffer was added to the first column of a clean 96 U-shaped plate, 25 μl DBS eluate was added and mixed thoroughly. 25 μl of the mixture was transferred to a second column and 25 μl sensitized particles were added to column one, 25 μl unsensitized particles to column two. Plates were covered and incubated for at least two hours at room temperature on a vibration free surface, before result interpretation. Discordant results between the two technicians were recorded as indeterminate. This protocol allows TPPA and HIV serological tests to be performed from one DBS spot.

### TPHA

Plasma aliquots were brought to room temperature and 25 μl were used to test for treponemal antibodies by TPHA (Lab21 syphilis TPHA, Lab21 healthcare, Kentford, UK). TPHA plasma tests were performed according to manufacturer’s protocols by trained laboratory technicians.

For DBS TPHA testing, the following protocol was developed; 25 μl of DBS eluate (obtained as described above) was added to 25 μl sample diluent, mixed and divided over two wells (25 μl each). 75 μl test or control cells were added and incubated for 1 hour. For both TPHA and TPPA protocols, the final DBS sample elution volume was kept comparable with plasma sample volume. Discordant results between the two technicians were recorded as indeterminate.

### EIA

Plasma aliquots were brought to room temperature and 50 μl were used to test for treponemal antibodies by Enzyme Immuno Assay (EIA) (Lab21 Syphilis Total Antibody EIA, Lab21 healthcare, Kentford, UK). EIA plasma tests were performed according to manufacturer’s protocols by trained laboratory technicians.

A DBS EIA protocol was developed in collaboration with the developer of the assay. 40 μl DBS eluate was added and incubated for two hours at 37°C. Plates were washed five times using an automated washer, 50 μl conjugate was added, shaken and incubated for 30 minutes at 37°C, washed five times, 50 μl substrate was added and incubated for 30 minutes at room temperature while kept in the dark. 50 μl stop solution was added and the wells were read as Optical Density (OD) 450/620 nm using an automated reader (DTX 800, Beckman Coulter, USA) with cut-off limits calculated according to the instruction manual. The results were then entered directly into the laboratory information management system.

### RPR

Quantitative RPR (BD Macro-vue RPR, Beckton Dickinson, Sparks MD, USA) was performed according to manufacturer’s protocols using plasma samples by trained laboratory technicians.

### Data analysis

The sensitivity, specificity and confidence intervals were calculated according to standard methods. The agreement between various methods was tabulated. Microsoft Excel (Microsoft, USA) and the statistical software Stata 11 (StataCorp LP, Texas, USA) were used for analysis of the results.

## Results

### Phase 1: Preliminary evaluation of different syphilis serological methods with DBS samples

During the first phase of the project, protocols for DBS samples were developed for TPPA, (DBS TPPA) TPHA (DBS TPHA) and EIA (DBS EIA). 464 DBS samples were tested with TPPA, TPHA, and EIA. Table 1 shows the sensitivity and specificity of all three syphilis serology tests using DBS samples compared to plasma samples. The DBS EIA was discontinued before the end of phase 1 because of the many false positive results (specificity 50.4%). The TPHA was also excluded from further testing because of low sensitivity (50.6%).

**Table 1:** Preliminary evaluation: performance of three syphilis serological assays using Dried Blood Spots compared to plasma

**Plasma as reference (using the same assay)**	**Positive samples detected**	**Negative samples detected**	Sensitivity (95% CI)^*^	Specificity (95% CI)^*^
**DBS TPPA**^**†**^**(n=463)**	82/96	363/367	85.4%	98.9%
			(76.7–91.8%)	(97.2–99.7%)
**DBS EIA (n=282)**	53/56	114/226	94.6%	50.4%
			(85.1–98.9%)	(43.7–57.1%)
**DBS TPHA**^**‡**^**(n=445)**	46/91	353/354	50.5%	99.7%
			(39.9–61.2%)	(98.4–100%)

n= samples.

^*^ 95% confidence interval.

^†^ 1 TPPA DBS indeterminate excluded.

^‡^ 19 TPHA indeterminate results excluded.

**Figure 2:**
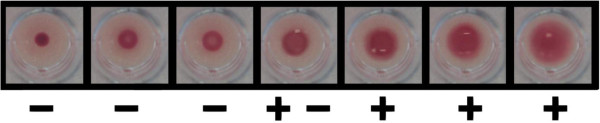
**DBS TPPA test result of seven patients.** Test outcome (− = negative, + − = indeterminate, and + = positive) was used in phase 2 of this study.

To improve sensitivity of DBS TPPA, technicians were trained to use a lower cut-off for interpretation of the DBS TPPA test results, based on the agglutination patterns seen in phase 1. Reading was adjusted by altering the agglutination positive and negative thresholds applied for DBS samples because of the higher background compared to plasma samples (Figure 2).

### Phase 2: Evaluation of DBS samples in comparison to matching plasma samples

For the final evaluation of DBS TPPA, 1,181 matching DBS and plasma samples were included. The average age was 31.9 years (range 15–84) and 760 participants were female (64%). For 8 persons, no data on gender or age was recorded and for another 3 persons no data on gender was available. Out of the 1,181 samples, 179 (15.2%) plasma samples tested positive by TPPA (Table 2). Excluding 34 indeterminate results, DBS TPPA showed a sensitivity of 95.5% (95% CI: 91.3–98.0%) and a specificity of 99.0% (95% CI: 98.1–99.5%) compared to TPPA plasma as the reference method. The DBS TPPA reading adjustments incorporated in phase 2 resulted in a 10.1% increase in sensitivity without compromising specificity. An overall agreement of 98.7% between the two readers was found. No non-specific reactivity (positive with unsensitized particles) with DBS samples was detected. Of the 34 indeterminate results for DBS TPPA, 30 (88.3%) were negative and 4 (11.7%) were positive for TPPA using plasma samples. Out of the 34 indeterminate results, 15 samples were deemed indeterminate because of discordant readings by two technicians and 16 samples were called indeterminate by both technicians. Of the 179 TPPA positive plasma samples, 66 were RPR positive (Table 3). DBS detected 66 out of 67 samples that were plasma TPPA and RPR positive. The one discordant result was DBS TPPA indeterminate. Unfortunately it was not possible to retest the 10 false positive DBS samples since insufficient sample eluate was available by the time plasma and DBS results were compared. To obtain the titration of the eight false negative DBS samples, matching plasma samples were retested with quantitative TPPA and quantitative RPR, as shown in Table 4. Four plasma samples were negative when retested with TPPA, suggesting a borderline sample or false positive reading when initially tested.

**Table 2:** **Correlation between detection of *Treponema pallidum *antibodies by plasma TPPA and DBS TPPA**

**(n=1147)**		**TPPA plasma**		
		**Positive**	**Negative**	**Total**
DBS TPPA	Positive	169	10	179
	Negative	8	960	968
	Total	177	970	1147*

sensitivity of DBS against plasma 95.5% (95% CI: 91.3–98,0%).

specificity of DBS against plasma 99.0% (95% CI: 98.1–99.5%).

* excluding 34 indeterminate results.

**Table 3:** RPR titres of TPPA DBS positive samples

**RPR titre**	**N=179**
Negative	113
1/1	13
1/2	19
1/4	13
1/8	7
1/16	4
1/32	6
1/64	1
>1/128	3

**Table 4:** Rapid Plasma Reagin (RPR) results on 8 plasma samples with false negative DBS TPPA results

**Sample**	**DBS**	TPPA^*^	**RPR**^**†**^
BBI99P	N	N	N
BBI792	N	N	N
BBI6GG	N	N	N
BBI5YI	N	N	N
BBI8ZC	N	1/80	N
BBI6LP	N	1/160	N
BBI6K6	N	1/320	N
BBI6NZ	N	>1/640	1/64

N= negative.

^*^ Quantitative TPPA.

^†^ Quantitative RPR.

## Discussion

We developed and validated protocols for the use of DBS samples with various syphilis serological assays. DBS samples can be recommended for use with TPPA, and may be of value for external quality assurance of point-of-care syphilis testing. When finger-prick blood has been obtained to perform syphilis POC testing, DBS can be used to collect blood for EQA purposes directly afterwards. Finger-prick blood spotted onto filter paper can be stored and shipped at room temperature, allowing the samples to be transported to a central laboratory where retesting can be done. The reduction in required materials, no cold chain requirements and minimal training of personnel at clinic level, decreases costs considerably in comparison to standard blood collection by venepuncture [16]. Additionally, the stability of human antibodies stored on DBS has been shown to be resilient to ambient temperatures for months [17-19]. This potentially makes DBS a suitable sample for EQA of POCTs in remote settings.

Unfortunately, we were not able to obtain acceptable performance for the use of DBS samples with the Syphilis Total Antibody EIA and Lab21 Syphilis TPHA. The EIA false positive results were primarily caused by a high background, possibly due to substances eluted from the filter paper and whole blood that adhered non-specifically to the wells. The TPHA false negative results were most likely caused by a reduced sensitivity when using DBS samples. DBS samples tested with TPPA gave a sensitivity of 95.5% and specificity of 99.0% compared to plasma samples.

Of the eight samples that were false negative by DBS TPPA, four were negative when retested with quantitative TPPA on plasma samples and four were false negatives, of which two had relatively high TPPA titres (1/320 and 1/640). We tested all plasma samples using the RPR assay to determine if any of the false negative samples were from women with active syphilis, defined as being RPR and TPPA positive. Since only one false negative DBS sample was positive for RPR, TPPA using DBS samples showed excellent sensitivity for the detection of active syphilis

It should be noted the study took place in Tanzania. Although tests were performed and stored according to the manufacturer’s recommendations, potential environmental effects by transporting and using the kits under tropical conditions could not be completely ruled out. Additionally, only one DBS spot was available per sample which restricted the ability to retest discordant test results or develop appropriate procedures for indeterminate samples.

Because TPPA is an agglutination assay, experience in reading results is essential and therefore training is necessary. 34 DBS samples (3%) were marked as indeterminate due to difficulty in interpreting the results or because of discordant reading by two technicians. DBS samples can potentially be used for quantitation with TPPA, although it would require evaluation against titers obtained with plasma samples. Because of the subjectivity, it is recommended that TPPA should be read by two readers.

DBS TPPA has been used as a surveillance tool in a few studies [9,11,12] that used a protocol developed by Coates *et al.*[12]. The DBS TPPA protocol developed in this study is an improvement to the protocol developed by Coates *et al.* as we adjusted the elution so that the sample input into the TPPA assays from DBS and plasma are comparable. We also included unsensitized particles in the procedure to control for biologically reactive samples.

## Conclusions

As prenatal screening for syphilis using POCTs becomes widely implemented, an EQA method appropriate for use with blood collected by a finger prick must be developed to assure the proficiency of POC testing in rural or remote areas. The objectives of this study were to develop and validate DBS protocols for use with commercially available syphilis diagnostic assays, and to determine their performance in syphilis serological assays using plasma samples as a reference. Based on the high sensitivity and specificity of DBS TPPA compared to plasma TPPA, DBS can be recommended for use with TPPA. Our study also showed the importance of training laboratory technicians in performing and reading the DBS TPPA, even when they are already trained in plasma TPPA. We obtained a 10.1% increase in sensitivity when technicians were more experienced in interpreting DBS TPPA agglutinations.

## Abbreviations

DBS: Dried blood spots; TPPA: *Treponema pallidum* particle agglutionation; TPHA: *Treponema pallidum* haemagglutination assay; EIA: Enzyme immunoassay; RPR: Rapid plasma reagin; VCT: Voluntary counselling and testing; OD: Optical density; POCT: Point of care test; QA: Quality assurance.

## Competing interest

The authors declare that they have no competing interests.

## Authors’ contribution

PWS initiated the study, developed laboratory protocols and drafted the manuscript. TV developed protocols and drafted the manuscript. DM and RWP provided supervision throughout the study and made major contributions to editing the manuscript. JT was responsible for sample collection, sample process and revision of the manuscript before submission. JM and JC provided supervision for laboratory work, handling and extraction of the data. BDC provided guidance on the data analysis and participated in the interpretation of results. AA, BZ and MU planned sample collection and collaborated in writing of the manuscript. All authors read and approved the final manuscript.

## Pre-publication history

The pre-publication history for this paper can be accessed here:

http://www.biomedcentral.com/1471-2334/13/102/prepub
